# The role of self-esteem in mental health: a comparative study on its association with anxiety, depression and stress in support-seeking and general populations from Croatia

**DOI:** 10.1192/j.eurpsy.2025.1846

**Published:** 2025-08-26

**Authors:** T. Meštrović, J. Mašek, R. Ribić, M. Neuberg

**Affiliations:** 1 Department of Nursing, University North, Varaždin, Croatia

## Abstract

**Introduction:**

Mental health is essential for overall well-being and resilience in facing life’s challenges. Self-esteem, a crucial mental health determinant, affects how individuals perceive and manage stress, and may be linked to worse health outcomes when conditions like anxiety and depression are concerned. Consequently, understanding the role of self-esteem in mental health is essential for developing effective preventive strategies and interventions.

**Objectives:**

In this study we aimed to investigate the relationship between self-esteem levels and anxiety, depression and stress, with a hypothesis that the presence of the aforementioned mental health challenges is significantly correlated with lower levels of self-esteem. We sought to explore the potential impact of self-esteem as both a risk factor and a target for interventions in mental health care.

**Methods:**

A cross-sectional study design was employed, involving 100 participants based in the Republic of Croatia and divided equally into two groups: support-seeking individuals or patients from the daily clinical setting and a control group with no psychiatric treatment history. Participants completed the Rosenberg Self-Esteem Scale (RSE) and the Depression Anxiety Stress Scale (DASS-21). The self-esteem scores classified individuals into low, average or high self-esteem categories. Statistical analyses, including chi-square tests and Mann-Whitney U tests, assessed differences in DASS-21 scores between groups, with adjustments for sociodemographic factors.

**Results:**

Psychiatric patients showed significantly lower self-esteem (mean = 20.84, SD = 7.161) than the control group (mean = 29.84, SD = 7.709). Additionally, psychiatric patients experienced higher levels of anxiety (mean = 12.60, SD = 5.237), depression (mean = 12.04, SD = 5.595) and stress (mean = 14.06, SD = 4.433) when compared to the control group, which exhibited mild anxiety (mean = 5.14, SD = 5.018), low depression (mean = 4.90, SD = 5.296) and minimal stress levels (mean = 6.96, SD = 4.973). Statistical analysis revealed a statistically significant association between low self-esteem and support-seeking or patient status (p = 0.001). Additionally, low self-esteem was associated with more severe mental health symptoms across all categories (p < 0.05).

**Conclusions:**

This study emphasizes the interconnectedness between self-esteem and mental health, demonstrating that lower self-esteem is associated with higher anxiety, depression and stress levels. Therefore, integrating self-esteem enhancement into therapeutic settings may be an effective strategy for mitigating mental health risks, providing a proactive approach to psychological care and prevention.

**Disclosure of Interest:**

None Declared

